# Pyrrho, on Apoplexy

**Published:** 1802-04

**Authors:** 


### *To the Editors of the Medical and Phyfical Journal.*

GENTLEMEN,

AS a friend to the Medical Journal, I have experienced no fmall fenfation of regret, in obferving fome late Numbers deformed by the virulence of perfonal hatred, a fpirit equally unworthy the name of a Philofopher, and hoftile to the advancement of Science and of Truth: I allude to the Communications upon Apoplexy, between Mr. Crowfoot and Dr. Langslow. This fenfation I have felt more keenly, knowing, from fome connection which I have with the eaftern counties of this kingdom, that Mr. C. is held in high and deferved efteem in his neighbourhood, and that his profeffional knowledge and integrity are well known in the medical community in which he lives. If fuch is the cafe, it is much to be lamented that your various Correfpondents, on this fubject, who are ftrangers to the parties concerned, fhould hazard opinions and judgments of the value of their characters, and propriety of their conduct; as by fuch a proceeding, the feelings of the liberal and enlightened may be unjuftly, though unintentionally, wounded.

The ftatement, as prefented to the public, certainly does not convey that information which is neceffary to form an accurate and candid judgment of the propriety of conduct of the refpective parties; for it is to be collected from that ftatement, that Mr. C's letter was not written for the public eye, and that it was printed without his knowledge or confent; it may alfo be obferved, that any difference of opinion between him and Dr. L. concerning the ufe of emetics in Apoplexy, was not the caufe of their difagreement, but fome previoufly exifting motives had determined Mr. C. againft an interview with Dr. L. as had been previoufly made known and mentioned to the hufband of the lady. It is poffible thefe motives may have juftified Mr. C. in refufing an interview.

I have made thefe remarks in the fpirit of candor, and in the hope that they may tend, in the future progrefs of this inquiry, to to recall the attention of the medical world from perſonal invective to the queſtion under conſideration; a queſtion of conſiderable magnitude, and deſerving the minuteſt inveſtigation.

Before I proceed, it muſt be here remarked that the queſtion is not, as ſeems to have been underſtood, whether bleeding or emetics are to be uſed in Apoplexy, for the uſe of blood-letting does not ſeem to have been denied; but whether emetics are at any time uſeful in Apoplexy? It is evident there are two ways of determining this queſtion; the one is by the weight of authority; the other is by reaſoning upon and determining the nature of the action of emetics upon the ſyſtem at large. And if we are to look to the tribunal of authority upon this matter, I muſt freely confeſs that it has been with no ſmall degree of ſurpriſe that I have obſerved Dr. L. raiſing ſo high the voice of triumph over his opponent; as if he ſtood alone and unprotected in his opinion, that an emetic may, at times, be uſeful in Apoplexy. Such an opinion, I truſt, has at leaſt the ſanction of authority, as will be proved in the ſequel; and he who would charge its abettors with ignorance, can be but little acquainted with the hiſtory of medical opinions.

I have no deſire to cite a long liſt of medical authors favoring the uſe of vomits in this diſeaſe; but it may not be foreign to the ſolution of the preſent Inquiry, to remark, that amidſt many it will be found that Riverius and Helmont, that Mayerne and Etmuller thought well of them; neither needs that man to bluſh who, on this ſubject, joins in opinion with the ſagacious the immortal Sydenham, the experienced Shaw, or the diligent Lieutaud. Indeed, ſo little acquainted ſeems Dr. L. with the opinions even of more modern authors upon the continent on this ſubject, that it is neceſſary to inform him that the judicious Selle, of Berlin, and the late Profeſſor Stoll, of Vienna, have actually made a triple diviſion of Apoplexy, referring one ſpecies of it to the ſtomach, and naming it gaſtrica from its ſeat; their diviſion is, therefore, into the ſanguinea, the gaſtrica, and the ſeroſa or maligna. It needs not be remarked, that in the gaſtrica, emetics are the principal agents of cure. I ſhall not ſwell this liſt of names any farther, enough having been ſaid to prove, that he who charges perſons holding ſentiments ſimilar and congenial with ſuch characters with ignorance, fails at leaſt in candor, if not in information.

I have dwelt ſufficiently long on the uſe of emetics, as warranted by authority, which, however, on medical queſtions, in oppoſition to Dr. L. I am ready to confeſs has exerciſed a controul ſo unlimited over the freedom of inquiry, as to have arreſted in many inſtances the progreſs of truth. I muſt moreover remark, that the vaunting manner in which Dr. L. has

ſpoken both of Mr. Crowfoot and Dr. Girdleſtone, in diſcuſſing this ſubject, ſeems but little authoriſed, from the very nature of the preſent inquiry; and feeble muſt be that mind, that is awake to any great ſenſe of merit and ſelf-gratulation, in merely being able to detail and put in force the common received notions in the treatment of a very common diſeaſe; and I have alſo very ſtrong doubts whether theſe gentlemen have retired from the field of controverſy, on this occaſion, from any ſenſe of their antagoniſt's ſuperiority, ſo much as from knowing that much doubt and obſcurity attach to the ſuppoſed advantage of any mode of practice in this diſeaſe; and from feeling that the temper in which the diſcuſſion has hitherto been carried on, is but little favorable to the developement of truth.

But to proceed. In turning from authority to the dictates of reaſon, in this matter, I would aſk, is it certain that from the action of a vomit upon the ſyſtem, there is an increaſed impetus or determination of blood to the brain? Or, ſuppoſing that there is an increaſed determination of blood, does it follow, neceſſarily, that a rupture of veſſels, or effuſion, would take place? It ſeems to me, in reflecting upon the effects of an emetic upon the ſyſtem, that the firſt circumſtance that aſſails the attention, is a great diminution or enfeebled action of the vaſcular ſyſtem in general; this is obſervable during the nauſea preceding the act of vomiting; in this ſtate there is great pallor of body, faint ſweats, the pulſe is feeble and unſteady, and the reſpiration is affected; evident marks of leſſened action in the heart and arteries. It will not therefore be aſſerted, by the enemies of emetics, that in nauſea the blood is ſent to the brain, either with greater force, or in greater quantity; on the contrary, it is reaſonable to ſuppoſe, that in this ſtate there is a ſmaller quantity of blood, and leſs forcibly, ſent to the brain. Hence, in this view, granting the exiſtence of fulneſs and diſtenſion of the veſſels of the brain, ſuch fulneſs, it is probable, might have time to be removed by the powers of the veſſels, while the blood, during nauſea, is ſent to that organ with leſs impetus, and in leſs quantity, from the diminiſhed arterial action. But it may be replied, if during nauſea there be diminiſhed arterial action, the ſame cannot hold good during the act of vomiting, in which the circulation of the blood is interrupted, and it is accumulated in the brain. But in this act I doubt whether this accumulation exiſts, and for the following reaſons: It is a well-eſtabliſhed fact that the motion of elevation and depreſſion peculiar to the brain, alternates with that of reſpiration; that is to ſay, that in expiration the elevation, and in inſpiration the ſubſidence, of the brain takes place.

Now

Now it being eſtabiſhed,* that the act of vomiting takes place rather in a ſtate of inſpiration, in which, from the free return of the blood through the heart and the lungs, the ſubſidence of the brain, and, of courſe, its diminiſhed vaſcular fulneſs happen, it ſeems probable, notwithſtanding the appearance of ſuffuſion and fulneſs of the face, that in the act of vomiting, the ſtate of the brain is rather that of depletion than plenitude; on this ground, therefore, I ſuppoſe, it may be doubted whether emetics are hurtful by increaſing preſſure on the brain.

And it may be farther obſerved in general, that as the blood is the proper ſtimulus and cauſe of action of the vaſcular ſanguiferous ſyſtem, and a ſtimulus by which its tone and denſity, as compoſed of living ſolids, are kept up and ſupported, it would ſeem that the ſtrength of any given veſſel or veſſels, is in a direct ratio of the force of that ſtimulus; or, in other words, of the momentum and velocity with which the blood moves; hence it appears improbable that an increaſed impetus of the blood ſhould rupture a veſſel; and that the appearance of blood or ſerum effuſed, depends upon other cauſes inherent in the veſſels of the brain, rather than upon the increaſed motion of the blood. This poſition receives conſiderable illuſtration from anatomical experiment; it does not happen by throwing a ligature over the principal artery leading to a part, or over a principal vein leading from a part, that ſuch a veſſel ſo treated is ever ruptured, or that the ſmaller arteries and veins in the neighbourhood, by having an undue quantity of blood thrown upon them, give way or are ruptured; and it is known, that by ſuſpending animals by a rope thrown round their necks, it is a difficult matter to kill them, providing an opening is made in the trachea, below the rope, notwithſtanding the preſſure upon the veſſels, and which is probably more felt by the veſſels carrying the blood from the brain, than the arteries leading to the brain. Theſe facts prove at leaſt that it is not common to rupture veſſels by increaſing the impetus of the blood.

This poſition receives farther illuſtration from reflecting upon ſome morbid phenomena, in which the action of emetics would be ſuppoſed to excite hæmorrhage, if ſuch were the natural or neceſſary effects of ſuch action. It is well known that emetics are frequently and repeatedly employed in diſeaſed ſtates of the lungs, and where a diſcharge of blood has previouſly and repeatedly taken place, and that in ſuch caſes the appearance of hæmorrhage is retarded rather than promoted by their uſe. And there have alſo been caſes † of perſons la-

* Vide Soemmering de Corporis Fabrica.

† Vide Heberden's Commentarii de Morborum Hiſtoria.

bouring under frequent and alarming hæmoptyſis, who have been ſent to ſea for their health, and who have never paſſed a day for months without ſtrong efforts to vomit, and yet without any recurrence of hæmorrhage. And I have alſo known two caſes of perſons contending with epiſtaxis, in an unuſual and highly threatening degree, and in which circumſtances have made it neceſſary, fearing a fatal change, to remove the patients to about a mile's diſtance; and in theſe caſes, in which perfect reſt and cool air would have been enjoined as neceſſary, and in which the motion of the carriage would have been thought likely to increaſe the diſcharge; I ſay, contrary to expectation, and the fiat of authority, it has been finally put a ſtop to while the carriage was in motion.

Theſe conſiderations ſeem, at leaſt, to render it doubtful, whether even in caſes where hæmorrhage has taken place, and where there has been a ſuppoſed tendency to rupture of veſſels by increaſing the impetus of blood, ſuch tendency is not rather diminiſhed than increaſed, by the increaſed tone and denſity of the veſſels from ſuch impetus; and they alſo ſeem to ſhew that emetics, if hurtful in Apoplexy, prove ſo from ſome other mode of action than that of determining to the head.*

I ſhall here diſmiſs the queſtion of emetics, and ſhould have proceeded immediately to the conſideration of Apoplexy, as at all times dependent upon the effuſion of blood or ſerum in the brain; an aſſertion haſtily hazarded by Dr. L. and, as appears to me, at variance with ſound analogy and the evidence of the beſt medical records: But before I proceed, I can but remark, that I think Dr. L. a little unfortunate at his attempts at ridicule, and to have proved at leaſt, in oppoſition to ſome elegant writers, that it is not a teſt of truth; for having ridiculed a ſtate of Apoplexy as connected with the ſtomach, he proceeds through the ſmall and large inteſtines, and at length fixes his ſhafts in triumph, in the toes. Now this ſituation, in my opinion, is ſomewhat out of ſeaſon and unfriendly; for I am willing to hope that Dr. L. is not ſo totally ignorant of the opinions of authors, even modern, as not to know that one ſpecies of Apoplexy has been thought to have ſome connection with the toes, the moſt common ſeat of gout; and hence Burſerius in his Inſtitutiones Practicæ, has given us an Apoplexy a metaſtaſi materiæ arthriticæ.

Dr. L. will excuſe me, if I point out another inconſiſtency in his reſolutions, if not in his reaſoning; for with great anxiety for the promotion of medical knowledge, and of that of the

---

* That which P. has admitted, p. 330, the impediment to the return of the blood from the head. Ed.

the difeafe in queftion, he has refolved upon a publication upon the fubject, to facilitate to the young and inexperienced, a diftinction in his opinion of the greateft importance in practice; that is to fay, the mode of diftinguifhing fanguineous and ferous Apoplexy. Now, at the fame time that he holds forth the importance of this diftinction, he confeffes that blood-letting is not only neceffary in fanguineous, but in ferous Apoplexy. This being allowed, it is difficult for me to difcover the very great importance of the diftinction; for if difeafes thought to be fo different are to be treated by the fame means, it will require no common logic to prove the neceffity of the knowledge of fuch difference. I wifh thefe remarks to be confidered as applying to Dr. L's fenfe of the neceffity of fuch knowledge, and not as in any degree advancing any opinion concerning fuch diftinction.

Perhaps, it may relieve Dr. L. from his folicitude in publifhing, when he is told, that even Morgagni has admitted bleeding in ferous Apoplexy; and from a conviction that the diftinction referred to is not fo neceffary, he may be alfo told, that Portal has lately publifhed in France, two Memoirs on Apoplexy, the tendency of which is to prove, that the diftinction, which Dr. L. thinks fo important, is not only futile, but fatal, as, in his opinion, both forms of Apoplexy require bleeding and a fimilar treatment. And I may alfo mention, that Hippocrates and the other ancient phyficians had no diftinction of this nature, dividing Apoplexy only into the levis and fortis.

I fhall now proceed to lay before your readers, and Dr. L. fome doubts which have arifen in my mind, from reflection, experience, and reading, on the exiftence of effufion in all ftates of Apoplexy. And being aware, that Dr. L. pays the greateft deference to the authority of writers on the fubject, I will take the liberty of introducing to his acquaintance, a few names fupporting the opinion, that Apoplexy has exifted without effufion. Thefe writers, I flatter myfelf, even Dr. L. will confefs are adequate judges of real Apoplexy; and he will not, I think, either as a philofopher or a phyfician, be afhamed to hold opinions confimilar with them. The perfons I allude to, are, the natural hiftorian and phyfician Vallifnerij, who has given feveral diffections of perfons dying apoplectic; and where, upon examination, to the furprize of every bye-ftander, effufion of no kind, nor any morbid appearance of the brain, prefented itfelf. Alfo I beg leave to cite to the fame end, Valfalva and Morgagni,* both of whom have acknowledged the

* Vide Adverfaria.

the exiftence of Apoplexy without effufion, or any morbid appearance of the brain.

I have alfo feen two cafes of perfons of very fpare habits, about 60 years of age, where there was not the leaft appearance of redundance of fluids, either ferous or fanguineous, in the fyftem, and who died ftrongly apoplectic. And moreover, if to the above facts we take into confideration the advanced period of life in which Apoplexy, for the moft part, takes place, we fhall find perhaps, in the natural hiftory of old age, fome changes and phenomena favorable to the generation of that diminifhed, nervous, or fenforial power, which forms the leading character of Apoplexy. In old age, it is a fact well eftablifhed, that there takes place throughout the folids of the body, a diminution of vafcularity, or, in other words, a converfion of living into fimple folid; thus, in this feafon of life, the bones and mufcles as well as other parts abound with fewer veffels; and this diminution of vafcularity becomes greater, the greater the age. Now if this be admitted, the fame changes which take place in other parts will alfo have their influence in the brain; hence in that organ, from a diminifhed vafcularity, and of courfe fenforial power, or from a converfion of living into fimple folid, a torpor is generated, favorable to that eclipfe of nervous power or fenfibility in which Apoplexy confifts. And this lofs of vitality of the brain appears to me a more probable caufe of Apoplexy, than any preffure from fluids either effufed or accumulated in the veffels. I have already, in fpeaking of emetics, attempted to fhew, that a rupture of the veffels of the brain is lefs likely to take place from any increafed diftention or impetus of blood, than from a weakened ftate or tone of veffels, from a weakened circulation or diminution of impetus; fimilar to what takes place in the ftate of veffels in fcurvy, and in the petechia and vibices of fome fevers. Dr. L. alfo, in fpeaking of the diftending power of the blood and the confequent rupture and effufion of fluids, feems to reafon from the old tenet of the fchools, that the volume of the blood is equal to, and in fome cafes even greater than the fum of the cavities of the veffels, and on that account he fuppofes injurious by over diftending and occafioning rupture or effufion; but let Dr. L. recollect, that in no inftance either in health or difeafe is the volume of the blood equal to the collective areas of the veffels; for upon killing any animal, in the fulleft and beft health, it will be found that the arteries are wholly empty, and that the blood in the fyftem will barely fill the veins; upon this view then, effufion cannot arife from over-diftention, neither is the indication for bleeding to be derived from that caufe. It muft alfo be confeffed, by every candid inquirer, that in diffections of the human body, no problem is more difficult of folution than that of determining what appearances are the precurfors or caufes of difeafe, and what are the fequelæ or effects. I would alfo afk in diffecting apoplectics, granting that in the brains of feven out of ten there is found an effufion of blood or ferum, or of both, are there no circumftances attendant upon the corpfes of apoplectics favorable to fuch appearances? Obfervation and reafon tell me, there are fome peculiarities, in fuch bodies, favorable to fuch effufion, as fequela or effects of difeafe: For in the bodies of apoplectics, within twenty-four hours after diffolution, there is for the moft part obfervable a great evafation of fluids of the body, which following the general law of gravity, fettle to the loweft parts; hence it happens, that in examining fuch bodies, there will be found a great collection even of extravafated blood along the neck, the fhoulders, the back, the nates, and the thighs; thefe parts, from the horizontal pofition, being undermoft. And if the veffels in fuch bodies have fo little power of retaining the fluids in thofe parts, what juft reafon can be affigned for the veffels of the brain having a greater power? And why, in that organ, fhould not a fimilar effufion take place after death, from the law of gravity acting upon the fluids? This effufion, it may be remarked, fhould take place in the brain in a greater ratio, that vifcus being fuppofed to contain a tenth of the blood of the whole fyftem, a greater portion than is contained, perhaps, in any other equal part.

Thefe confiderations have awakened in my mind, ftrong doubts of the exiftence of fulnefs of veffels or of effufion in the brain as the caufe of Apoplexy; and doubts, whether fuch appearances are not with more reafon to be confidered as effects.

But let it be conceded to Dr. L. that effufion either of blood or ferum does actually exift as a general caufe of Apoplexy; there is ftill a fmall difficulty awaiting this fuppofition, which he does not feem aware of, and which is, where are the veffels of the brain by which fuch effufed fluid is to be abforbed? Where are the lymphatics? And who has pointed them out either by the faithful teft of injection, or by appealing to the fenfes by diffections that cannot be doubted or denied? An anfwer to thefe queftions is important to Dr. L.: becaufe, whether five ounces, or five tea fpoons full of fluid be thrown upon the brain, it muft unceafingly remain there, unlefs thefe queftions can be duly anfwered.

On this fubject I have attended to the writings of a Haller and a Hewfon, of a Cruickfhank and a Monro, of a Caldani, a Mafcagni, and a Lupi; and I can find in them nothing fatisfactory on this head, or little more than idle conjectures and vague analogies.

This being the cafe, it has been matter of much wonder with me, that Dr. L. ſhould have made the abſorption of ſerum or blood effuſed in the brain (an organ not known to poſſeſs abſorbents) ſo eaſy and ſo quick a proceſs; while, for the moſt part, in other organs, and poſſeſſing abſorbents, it is a proceſs requiring conſiderable time.

Theſe arguments are ſufficient, at leaſt, to render uncertain the exiſtence of preſſure of the brain, from the effuſion of blood or ſerum, as a general and neceſſary cauſe of Apoplexy; and perhaps they throw ſome doubt upon the utility of blood-letting in this diſeaſe; which is, I think, farther confirmed by the following remark, That I have ſeen many caſes of Apoplexy, moſt of which have been treated by venæſection; but I confeſs, I have ſtrong doubts, from a compariſon of them, whether thoſe that have loſt the greateſt quantity of blood have not proved the moſt fatal.

Laſtly, I ſhall beg leave to ſuggeſt to your readers and Dr. L. that it is probable the ſeat of the cauſe of Apoplexy may be frequently placed in the lungs,* rather than the head. From the advancement of Phyſiology, and a more intimate knowledge of reſpiration, a perpetual renovation of the air in the lungs is a proceſs eſſential and neceſſary to life, and to the action of the heart and arteries; as by this proceſs animal heat is generated, and oxygen is conveyed to the ſyſtem at large, a principle widely diffuſed in the animal œconomy, and acting an important part on the irritable and ſenſorial ſyſtems. Now if it can be admitted, in old age, the ſeaſon of Apoplexy, that the lungs become leſs pervious to this principle, and leſs ready conductors of it to the ſyſtem, it may thence follow, that Apoplexy, or diminiſhed irritability or ſenſorial power, may take place. This ſeems, in a degree, confirmed by writers, who have at times connected the frequency of Apoplexy with certain ſeaſons or ſtates of the atmoſphere; as may be ſeen from Cole, Etmuller, and others; and alſo from the hiſtory of a family deſtroyed by the fumes of charcoal, as mentioned by Portal; in whom, upon diſſection, not only diſtenſion of veſſels had taken place upon the brain, but there was effuſion, as in *true Apoplexy.*

To conclude, the table of doubts here preſented to the public eye, is the reſult of my reading and reflecting upon this ſubject; and permit me to ſay, that I have little ſolicitude or anxiety about the reception of thoſe doubts; for ſhould they be removed and refuted, the cauſe of truth will be ſerved by the removal of error. And I ſhall be ſufficiently repaid for compoſing

* We ſhould wiſh to preſent our readers with ſome inſtance of this. Ed.

pofing this table, if by fhewing, that confiderable fhades of doubt and uncertainty hang upon this fubject, I fhall have repreffed in the Mover of this Controverfy, boldnefs of affertion and acrimony of invective; and fhall have begotten in him temperance and forbearance, in the inveftigation of this fubject, in which all parties muft confefs, there is much difficulty and uncertainty. And from a fenfe of this uncertainty, I can but regret, that I cannot accord with Dr. L. in his opinion, that Apoplexy is to be removed with a facility equalling in power the fafcination of a Mefmer, or the enchantment of a Perkins; and I muft candidly confefs, that it is to be doubted, whether medicine has ever done much towards its removal, whether it has been treated by bleeding or vomiting, by purging or blifters; and I feel no reluctance in admitting the truth of the axiom of Hippocrates, when he fays, "Apoplexiam folvere vehementem impoffibile; debilem non facile." And on this occafion, perhaps, may be applied to the human body, a remark which the fagacious hiftorian Tacitus has applied to the political, and who fays, "Cauta potiùs confilia cum ratione fequenda; quam profpera interdum, ex mero cafu fortuito."

PYRRHO.

